# Visualization of cytoplasmic organelles via in-resin CLEM using an osmium-resistant far-red protein

**DOI:** 10.1038/s41598-020-68191-z

**Published:** 2020-07-09

**Authors:** Isei Tanida, Soichiro Kakuta, Juan Alejandro Oliva Trejo, Yasuo Uchiyama

**Affiliations:** 1 Department of Cellular and Molecular Neuropathology, Juntendo University Graduate School of Medicine, Tokyo, Japan; 2 Laboratory of Morphology and Image Analysis, Research Support Center, Juntendo University Graduate School of Medicine, Tokyo, Japan

**Keywords:** Fluorescence imaging, Imaging, Optical imaging

## Abstract

Post-fixation with osmium tetroxide staining and the embedding of Epon are robust and essential treatments that are used to preserve and visualize intracellular membranous structures during electron microscopic analyses. These treatments, however, can significantly diminish the fluorescent intensity of most fluorescent proteins in cells, which creates an obstacle for the in-resin correlative light-electron microscopy (CLEM) of Epon-embedded cells. In this study, we used a far-red fluorescent protein that retains fluorescence after osmium staining and Epon embedding to perform an in-resin CLEM of Epon-embedded samples. The fluorescence of this protein was detected in 100 nm thin sections of the cells in Epon-embedded samples after fixation with 2.5% glutaraldehyde and post-fixation with 1% osmium tetroxide. We performed in-resin CLEM of the mitochondria in Epon-embedded cells using a mitochondria-localized fluorescent protein. Using this protein, we achieved in-resin CLEM of the Golgi apparatus and the endoplasmic reticulum in thin sections of the cells in Epon-embedded samples. To our knowledge, this is the first reported use of a far-red fluorescent protein retains its fluorescence after osmium staining and Epon-embedding, and it represents the first achievement of in-resin CLEM of both the Golgi apparatus and the endoplasmic reticulum in Epon-embedded samples.

## Introduction

CLEM (Correlative light-electron microscopy) is a method to correlate fluorescent images with electron microscopic images^1^. In traditional CLEM, fluorescent images of cells are first obtained after chemical fixation of the cells with paraformaldehyde and/ or glutaraldehyde. After acquisition of fluorescent images, the cells are treated with osmium tetroxide to provide contrast to the electron microscopic images. The cells are further dehydrated with a graded series of ethanol, and embedded in epoxy resins^2^. After the preparation of thin sections of samples embedded in epoxy resins, electron microscopic images of the thin sections are obtained. Therefore, these chemical modifications and the physical sectioning distort the sample’s morphology during the sample preparation for an electron microscopy. This represents an unavoidable limitation in traditional CLEM.

Another limitation is the Z-axis resolution of fluorescent images^3^. The X–Y axis resolution of a confocal microscopy is about 180–250 nm, while its Z-axis resolution is about 500–700 nm. In electron microscopic analysis, the thickness (i.e., its Z-axis resolution) of thin sections is about 50–100 nm. The difference in the Z-axis resolution of super-resolution fluorescence microscopy and that of electron microscopy is a serious constraint for correlating fluorescence and electron microscopic images.

One of the most efficient ways to overcome these limitations is to obtain both fluorescent and electron microscopic images from the same section following embedding in epoxy resins. The fluorescent intensity of most fluorescent proteins, however, is significantly weakened during post-fixation using osmium tetroxide, dehydration, and Epon embedding. These preparations are essential in order to preserve the membranous structures.

Technical approaches for limiting the reduction of fluorescence intensity during in-resin CLEM processing include: avoiding chemical fixation with osmium tetroxide and/ or to using other resins instead of Epon resins. Based on high pressure freezing and freeze substitution techniques, in-resin CLEM was established with standard fluorescent proteins and Lowicryl HM20 resins^4–7^. In these cases, standard fluorescent proteins (mGFP, mVenus, mRuby2, and YFP) are available, but special instruments and special resins are required.

Other approaches are to find a fluorescent protein that retains fluorescence after osmium staining and Epon embedding. Photoactivatable fluorescent proteins, mEos4a and mEos4b, are proteins reported to retain fluorescence after osmium staining^8^. However, the fluorescence is diminished during sample dehydration and Epon embedding. Recently, mEosEM, a variant mEos4b, was reported as a photo-convertible fluorescent protein that retains green fluorescence even after osmium staining and Epon embedding. This new protein has been used to accomplish in-resin CLEM of mitochondria and lamin-localized nuclear structures^9^. In-resin CLEM of other organelles, including the Golgi apparatus and the endoplasmic reticulum (ER) in Epon-embedded cells has not been achieved.

In this study, we show that a far-red fluorescent protein, mKate2^10^, retains fluorescence after osmium tetroxide staining and Epon embedding, and report the achievement of in-resin CLEM of the Golgi apparatus and ER in Epon-embedded cells using this protein.

## Results

### Fluorescence from mKate2 is preserved after osmium tetroxide staining and Epon embedding

**Figure 1:**
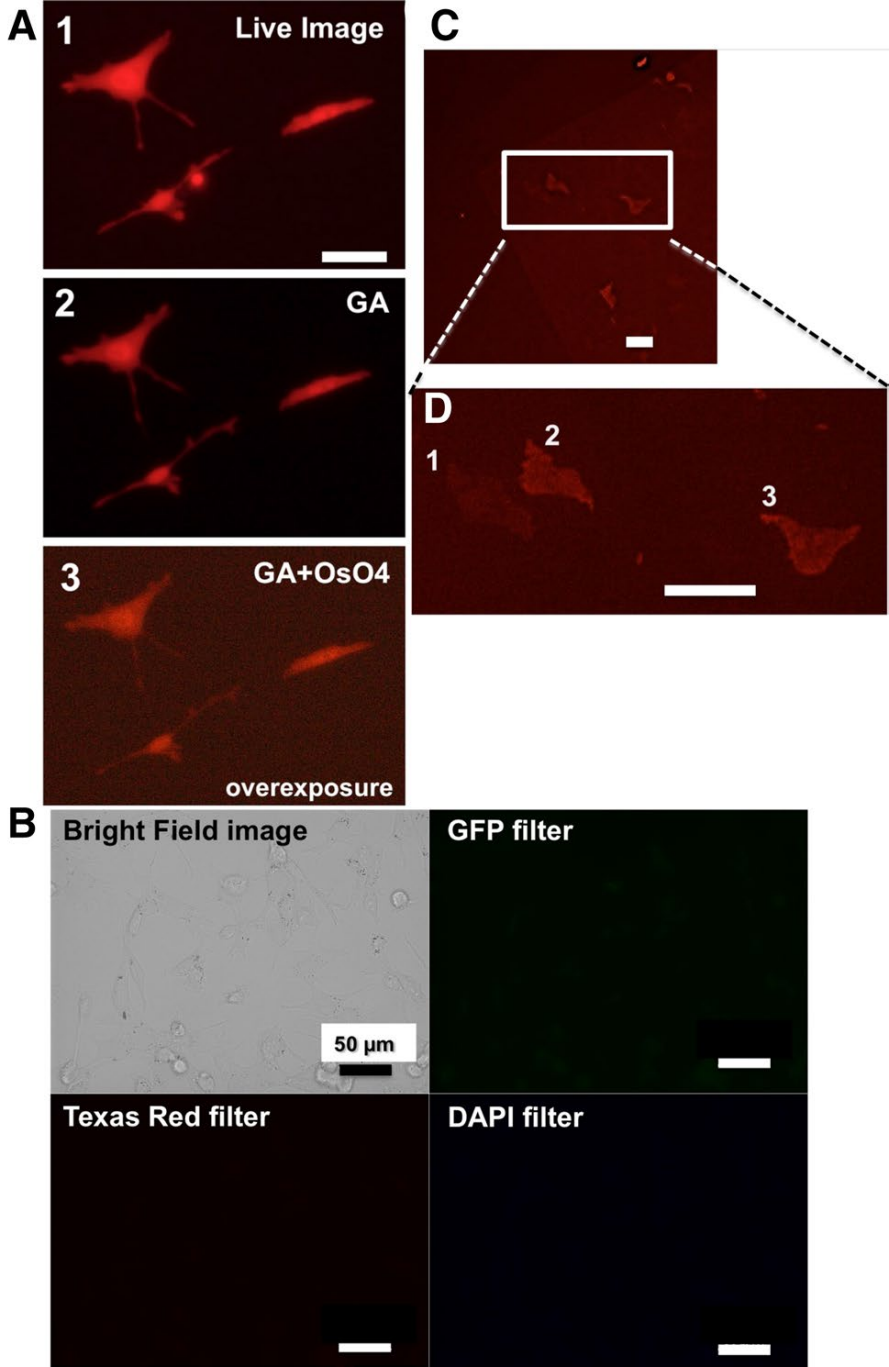
A far-red protein, mKate2, is suitable for in-resin CLEM of osmium-treated and Epon-embedded cells. (**A**) Fluorescence of mKate2 in HeLa cells after chemical fixations of glutaraldehyde and osmium tetroxide. HeLa cells expressing mKate2 (1, live image) (signal-to-noise ratio (mean ± SD) = 28.25 ± 3.18 dB; signal-to-background ratio = 8.14 ± 0.74) were pre-fixed with 2.5% glutaraldehyde (2, GA) (signal-to-noise ratio = 30.82 ± 1.96 dB; signal-to-background ratio = 8.07 ± 3.33), and post-fixed with osmium tetroxide (3, GA + OsO_4_) (signal-to-noise ratio = 28.81 ± 0.97 dB; signal-to-background ratio = 1.50 ± 0.17). The fluorescent images were obtained with a CKX530 cell culture microscope (Olympus) and a VisualixPro2 mertics CCD camera (KENIS) using a Texas Red filter. The fluorescent image in **c** was exposed about 20 times longer than those in 1 and 2. (**B**) Minimal levels of autofluorescence in HeLa cells after chemical fixations with glutaraldehyde and osmium tetroxide. The fluorescent images were obtained with a BZ-X710 fluorescence microscope (Keyence) using GFP, Texas Red, and DAPI filters (CCD monochrome camera, NIKON CFI60 series × 10 lens, gain + 2 dB). The signal-to-background ratios of the images from “GFP filter”, “Texas Red filter”, and “DAPI filter” are 1.29 ± 0.24, 1.12 ± 0.16, and 1.13 ± 0.18, respectively. (**C**) Fluorescent image of mKate2 in HeLa cells in a 100 nm thin section of Epon-embedded samples. The fluorescent image was obtained with a BZ-X710 fluorescence microscope (Keyence) using a Texas Red filter (CCD monochrome camera, NIKON CFI60 series × 10 lens, gain + 4 dB, 2 × 2 on chip binning). (**D**) High magnification of the area with a white square surrounding fluorescent image in C. The fluorescent image was obtained with a BZ-X710 fluorescence microscope (Keyence) using a Texas Red filter (CCD monochrome camera, NIKON CFI60 series × 40 lens, gain + 4 dB, 2 × 2 on chip binning). Scale bars, 50 µm.

We first investigated whether or not the fluorescence from mKate2 was preserved following prefixation with glutaraldehyde and postfixation with osmium tetroxide, which are procedures that are necessary for processing electron microscopy samples. We started by confirming the fluorescent protein expression in HeLa cells. These cells were fixed in 2.5% glutaraldehyde at 4 ºC for 1 h, and post-fixed in 1% osmium tetroxide at 4 ºC for 15 min. After the fixation process, the fluorescence emitted by mKate2-expressing cells was detected using a Texas Red filter (Fig. 1A). The exposure time necessary for the detection of fluorescence following chemical fixation was about 10–20 times longer than before chemical fixation. Under these conditions, the autofluorescence derived from cell treatments with glutaraldehyde and osmium tetroxide was negligible (Fig. 1B) and had no impact on the detection of mKate2 fluorescence using a Texas Red filter (Fig. 1A).

If the fluorescence from mKate2 could still be detected following dehydration with a graded series of ethanol and Epon embedding, the application of in-resin CLEM would be viable. Following chemical fixation with glutaraldehyde and osmium tetroxide, cells were dehydrated with a graded series of ethanol, and embedded in Epon resins at 60 ºC for 72 h. We sectioned 100 nm-thick Epon-embedded samples and placed them on glass cover slips. We used fluorescence microscopy to detect the fluorescence from mKate2 in the thin sections (Fig. 1C, D). These results indicated that the fluorescence from mKate2 was preserved in the Epon-embedded specimens following osmium tetroxide staining. Under these conditions, the overexpression of mKate2 hardly affected the intracellular structures in the cells (Supplementary Fig. 1). We detected only a few differences in the morphology of the organelles including nuclei, mitochondria, and profiles of the ER between fluorescence-positive cells and their neighboring fluorescence-negative versions in the electron microscope images.

### In-resin CLEM of mitochondria in Epon embedded cells

**Figure 2:**
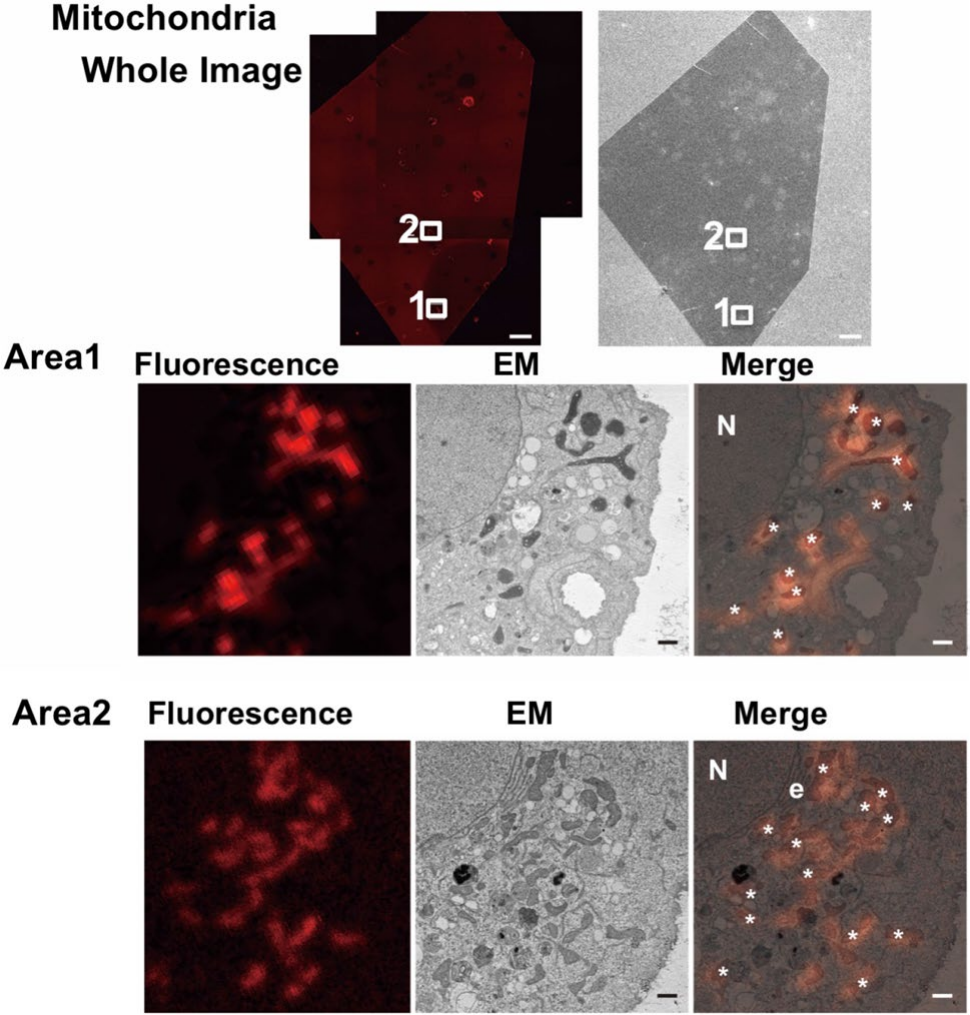
In-resin CLEM of mitochondria. The “Whole Image” indicates the whole images obtained from a LSM880 confocal fluorescence microscope and from a Helios NanoLab 660 scanning electron microscope (a backscattered electron detector at a voltage of 2.0 kV with a current of 0.4 nA). Scale bars, 100 µm. The images in Areas 1 and 2 indicate a magnification of the areas (white squares 1 and 2). Scale bars, 1 µm. The “Merge” is a merged image of the fluorescence image with an electron microscopic image (EM). “N”, “e”, and asterisks in the images indicate respective nucleus, endoplasmic reticulum, and mitochondria. Settings of a LSM880 confocal fluorescence microscope are as follows; bit depth, 8 bit; scan zoom, 2 × 2; pixel time, 8.19 µs; averaging, 2; Plan-Apochromat 63x/1.4 Oil DIC M27; beam splitter, MBS:MBS 488/561/633; DBS1, mirror; lasers 561 nm: 10%; excitation 561 nm; emission, 602 nm; detection wavelength 562–642 nm; detector gain 884.0.

To investigate the possibility that this protein could be suitable for accomplishing in-resin CLEM of mitochondria, we generated a plasmid for its expression tagged with the mitochondria-targeting signal of the *ActA* gene of *Listeria monocytogenes*^11,12^. We prepared Epon-embedded samples of cells expressing mitochondria-localized mKate2 (Supplementary Fig. 2), and analyzed them in thin sections using fluorescence microscopy and scanning electron microscopy. Images obtained by fluorescence microscopy show that fluorescent signals were preserved (Fig. 2). Positive signals for mitochondria in fluorescence microscopy were confirmed as mitochondria in corresponding cells via scanning electron microscopy (Areas 1 and 2 in Fig. 2). The CLEM images showed that the fluorescent signals correlated well with the mitochondrial structures in the electron microscopic images.

### In-resin CLEM of the Golgi apparatus in Epon-embedded cells

**Figure 3:**
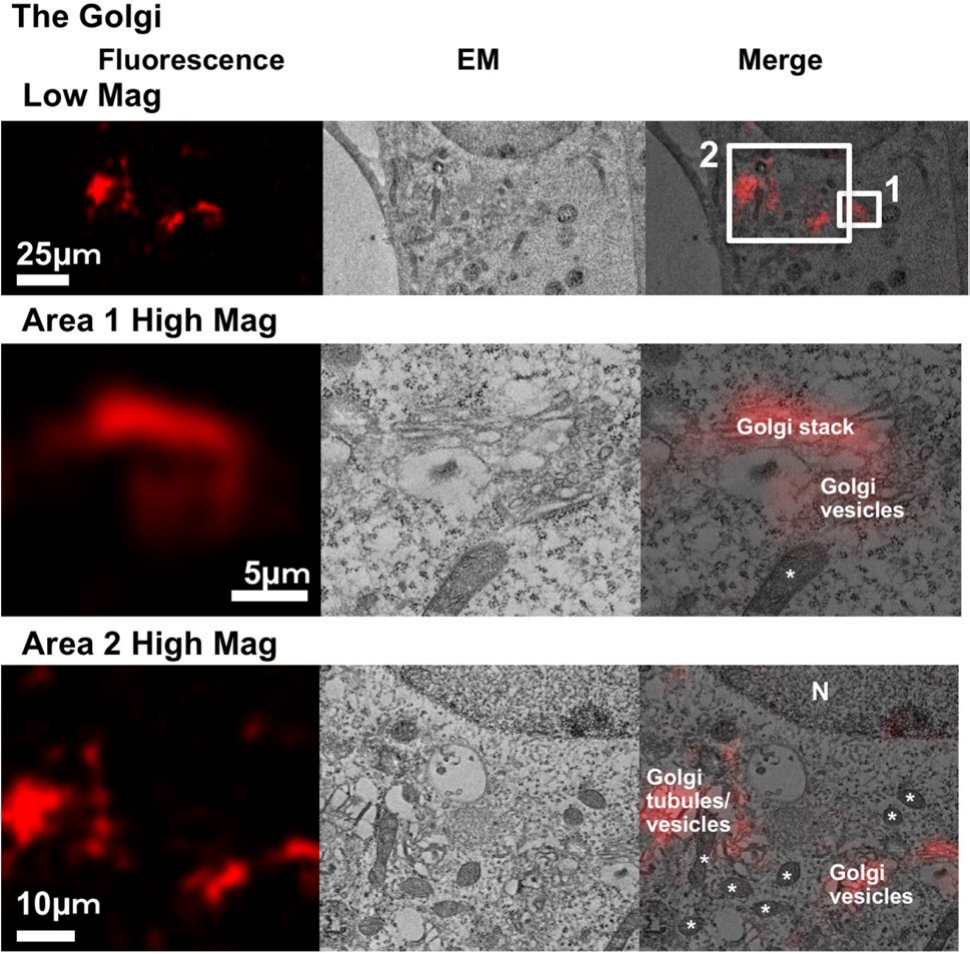
In-resin CLEM of the Golgi apparatus. The fluorescent image was obtained using a BZ-X710 fluorescence microscope (Teas Red filter, CCD monochrome camera, NIKON CFI60 series × 100 lens, gain + 4 dB, 2 × 2 on chip binning, haze reduction), and the electron microscopic image was obtained using a HT7700 transmission electron microscope. The “High Mag” in Areas 1 and 2 indicates a magnification of the areas surrounded by white squares 1 and 2 in the “Low Mag” areas. “N” and asterisks in the images indicate respective nucleus and mitochondria.

There is no current report of the in-resin CLEM of the Golgi apparatus in Epon-embedded specimens, and identification of Golgi-related vesicular/ tubular structures is difficult using electron microscopy. Therefore, if in-resin CLEM could be used for recognizing Golgi related structures, it would represent a great advance for the identification of intracellular organelles. To determine the suitability of mKate2 for the in-resin CLEM of Golgi-related structures, mKate2 was tagged with the Golgi marker β-1,4-galactosyltransferase (Supplementary Fig. 2). This new construct was expressed in cells that we used for performing in-resin CLEM of the Golgi apparatus (Fig. 3). Fluorescent signals (Fig. 3) were observed in thin sections via the use of fluorescence microscopy. Electron microscopic analyses revealed that fluorescent positive regions corresponded well with Golgi-cisternae (Fig. 3, Area 1) and with vesicular/tubular structures (Fig. 3, Area 2). These results indicated that the in-resin CLEM of Golgi-localized mKate2 is useful for the identification of fluorescent-positive Golgi-related vesicles and tubules in addition to representative Golgi stacks.

### In-resin CLEM of the endoplasmic reticulum in Epon-embedded specimens

**Figure 4:**
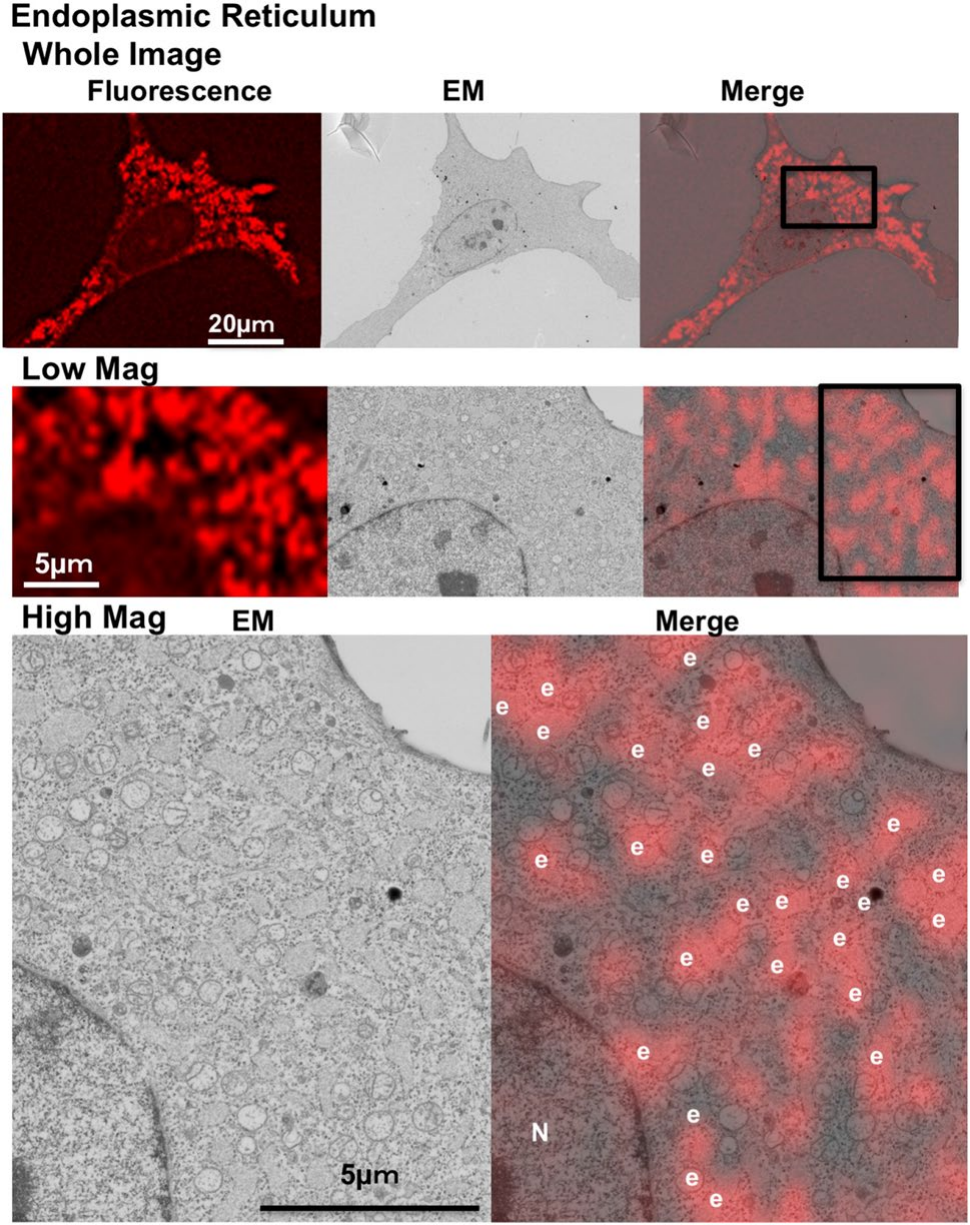
In-resin CLEM of the endoplasmic reticulum. The “Whole Image” indicates the whole images obtained from a BZ-X710 fluorescent microscopy and from a Helios NanoLab 660 scanning electron microscopy. The fluorescent image was obtained using a BZ-X710 fluorescence microscope (Teas Red filter, CCD monochrome camera, NIKON CFI60 series × 100 lens, gain + 4 dB, 2 × 2 on chip binning, haze reduction), and the electron microscopic image was obtained using a Helios NanoLab 660 scanning electron microscope (a backscattered electron detector at a voltage of 2.0 kV with a current of 0.4 nA). The “Low Mag” indicates a low-level magnification of the area surrounded by a white square in the “Whole Image”. “N” and “e” in the images indicate respective nucleus and endoplasmic reticulum.

No previous study has analyzed the ultrastructures of the ER in Epon-embedded cells using in-resin CLEM. In order to accomplish this, we expressed a fluorescent protein with an ER-targeting signal at the amino terminus^13^ and an ER-retrieve KDEL signal at the carboxyl terminus in cells^14,15^, and performed in-resin CLEM of the endoplasmic reticulum in the Epon-embedded cells (Supplementary Fig. 2). As shown in Fig. 4, in-resin CLEM revealed that fluorescence-positive signals are representative of the membranous structures of the ER with ribosomes in the electron microscopic image. The structures tended to swell slightly, which was likely due to an overexpression of this fusion protein.

## Discussion

In the present study, we found that a far-red fluorescent protein, mKate2, was available for the in-resin CLEM of Epon-embedded samples. This enable us to perform in-resin CLEM of the Golgi apparatus and the ER in Epon-embedded cells. As far as we could ascertain, this is the first report of a far-red fluorescent protein suitable for the in-resin CLEM of Epon-embedded specimens. The fluorescent signals of mKate2, mitochondria-, Golgi-, and ER-localized mKate2 proteins were detected in the thin sections of Epon-embedded cells. Representative ultrastructures of fluorescent protein-labeled organelles were observed via electron microscopy, which indicated that the fine structures were largely preserved. These results suggested that this fluorescent protein is suitable for the in-resin CLEM of intracellular membranous structures in Epon-embedded cells.

We performed in-resin CLEM of the ER in Epon-embedded cells. Epon embedding is one of most robust and essential treatments used to preserve the fine structures of the ER. ER membranes with ribosomes were recognized as fluorescent-positive signals in the in-resin CLEM of Epon-embedded samples. When ER-localized mKate2 protein was expressed in the cells, the fine profiles of the ER in the Epon-embedded cells tended to swell slightly, which likely was caused by the overexpression of ER-localized mKate2. In the case of the in-resin CLEM of mitochondria when using this protein, the mitochondria also tended to swell when the fluorescent signals were too strong. Therefore, fine structural changes in the mitochondria may require further attention.

We established that a far-red fluorescence emitted from mKate2 is preserved after osmium staining and Epon embedding. To evaluate the possibility that other fluorescent proteins might also retain fluorescence upon these chemical fixations, we examined other red fluorescent proteins including mCherry, mApple and mStable. mCherry is one of standard red fluorescent proteins derived from DsRed and mRFP^16^. mApple is brighter than mCherry^17^. mStable is a point mutant of mKate2 that increases the photostability of mKate2^18^. Unfortunately, these proteins were sensitive to chemical fixations with glutaraldehyde and osmium tetroxide, and only retained faint fluorescence when compared with that of mKate2 under our experimental conditions (Supplementary Fig. 3). Admittedly, it’s possible that these proteins might be available for in-resin CLEM of Epon embedded samples under different experimental conditions, since we only checked their retained fluorescence with a conventional Texas Red filter set. At present, there is no answer why mKate2 retains fluorescence after osmium staining and Epon embedding.

The sample preparation for this in-resin CLEM is similar to the standard preparation of samples for TEM^2^. Most cell biologists and histologists who rely on electron microscopy are familiar with these techniques. The in-resin CLEM of Epon-embedded specimens is enabled simply via the expression of mKate2 in the target cell, and no additional instruments or techniques are required for sample processing.

Another advantage of this protein is its fluorescent color. Recently, mEosEM was reported as a photo-convertible fluorescent protein resistant to osmium staining and Epon embedding^9^. The mEosEM protein exhibits green fluorescence in the epoxy resins. We compared the retained fluorescence of mKate2 with that of mEosEM after osmium staining (Supplementary Fig. 4). The retained fluorescence of mEosEM was detected with a GFP filter set, but not with a Texas Red filter set. In contrast, the retained fluorescence of mKate2 was detected with a Texas Red filter set, but not with a GFP filter set. Therefore, mKate2 is a potential second color for combining with mEosEM when performing two color in-resin CLEM of Epon embedded samples. In addition, a majority of the CLEM specialists use UV, green and middle-red channels. Therefore, researchers could adopt mKate2 as a new color channel alternative.

The next objective is the development of a multi-color in-resin CLEM of Epon-embedded samples that could be used to analyze the interactions among organelles and intracellular biomolecules. For this purpose, we currently are screening other fluorescent proteins. Furthermore, we are working with mKate2-based transgenic mice to investigate whether this method is applicable to mouse tissues.

## Methods

### Cells, media, and materials

COS1, HeLa and HEK293 cells were obtained from the American Type Culture Collection, and were cultured in Dulbecco’s Modified Eagle’s Medium (Wako, 045-30285) containing 10% fetal calf serum (JRH Biosciences/Sigma-Aldrich, 12603C). FuGENE HD transfection reagent was used to introduce the plasmid into cells (Promega, E2311). For the expression of the mKate2 and its fusion proteins, we used pCAGGS^19^, pEX^20^, pEXP^20^ and pAAV-CMV (TAKARA, 6673) plasmids. To generate an expression plasmid for mKate2 tagged with seven amino acids (GGGGSGL) at the carboxyl terminus, a DNA fragment (Supplementary Data) was generated by a KOD one PCR master mix (TOYOBO, KMM-201) using pEX-PK-hLC3 (Addgene #61458) as a template, and introduced into pCAGGS plasmid (pCAG-mKate2-G plasmid). For the expression of mitochondria-targeting mKate2 under the control of the CMV promoter, a DNA fragment encoding mKate2-fused with a mitochondria-targeting signal of the ActA gene of *Listeria monocytogenes* was introduced into pAAV-CMV (pAC-mKate2-mito)^11,12^. For the expression of Golgi-targeting mKate2 under the control of the CAG promoter, a DNA fragment encoding mKate2 tagged with a Golgi-targeting signal of β -1,4-galactosyltransferase 1 was introduced into pEX plasmid (pEX-mKate2-Golgi). For the expression of the ER-targeting of mKate2 under the control of the CAG promoter, a DNA fragment encoding mKate2 fused with an ER-targeting sequence of calreticulin and the ER retrieval sequence, KDEL^13^, was introduced into pEXP plasmid (pEXP-mKate2-ER)^14,15^.

### Sample-preparation, fluorescent microscopy, and electron microscopy for in-resin CLEM

Cells expressing mKate2 and proteins tagged with mKate2 were prefixed with 2.5% glutaraldehyde buffered in 0.1 N phosphate buffer, pH 7.2, at 4 ºC for 1 h. The fixed cells were washed twice with 0.1 N phosphate buffer, pH 7.2, and post-fixed in 1% osmium tetroxide at 4 ºC for 15 min. Fixed cells were incubated in TUK solution (Wako, 209-20851) at 4 ºC for 10 min when the fluorescence was significantly decreased. Cells were dehydrated with a graded series of ethanol, and embedded in Epon812 (Oken shoji) at 60 ºC for 72 h. Thin sections (100 nm) were cut with an ultramicrotome UC6 (Leica) and placed on glass cover slips that were coated with Pt/Au using an ion sputter E-1010 (Hitachi). Sections were observed in a TUK solution using either a BZ-X710 fluorescence microscope (Keyence) or a LSM880 confocal laser-scanning microscope (Zeiss). Thereafter, sections were stained with uranyl acetate and lead citrate, and observed via SEM (Helios NanoLab 660, FEI). The SEM Images were obtained using a backscattered electron detector (CBS detector) at a voltage of 2.0 kV with a current of 0.4 nA. When indicated, TEM images were obtained via TEM (HT7700, HITACHI). The sample transfer from fluorescence microscopy to TEM was performed as described previously^5^.

## Supplementary information

Supplementary Information (DOCX 4051 kb)

## Abbreviations

CLEM: Correlative light-electron microscopy
ER: Endoplasmic reticulum
SEM: Scanning electron microscopy
TEM: Transmission electron microscopy
