# Supplementary material for: Visualization of cytoplasmic organelles via in-resin CLEM using an osmium-resistant far-red protein
Source: Sci Rep. 2020 Jul 9;10:11314. doi: 10.1038/s41598-020-68191-z (PMC7347593; doi:10.1038/s41598-020-68191-z)
Supplement: Supplementary file 1 — Supplementary Information (DOCX 4051 kb) [file 41598_2020_68191_MOESM1_ESM.docx]

**Visualization of cytoplasmic organelles via in-resin CLEM using an osmium-resistant far-red protein**

Isei Tanida,^1*^ Soichiro Kakuta,^1,2^ Juan Alejandro Oliva Trejo,^1^ and Yasuo Uchiyama^1*^

^1^ Department of Cellular and Molecular Neuropathology, Juntendo University Graduate School of Medicine, Tokyo, Japan

^2^ Laboratory of Morphology and Image Analysis, Research Support Center, Juntendo University Graduate School of Medicine, Tokyo, Japan

***** Correspondence: tanida@juntendo.ac.jp (I.T.) and y-uchi@juntendo.ac.jp (Y.U.); Tel: +81-3-5802-1025 (I.T.)

**Supplementary Fig. 1. Little effect of overexpression of mKate2 on ultrastructures of intracellular organelles.** Electron microscopic images were obtained from the same thin section in Fig. 1D. The cells (1-3, black dotted enclosing lines in A and D) were identical to the cells expressing mKate2 in Fig. 1C (white dotted enclosed lines). B and C are higher magnification images of squares I and II accordingly, in A. E and F is a higher magnification of the white squares in respective D and E.


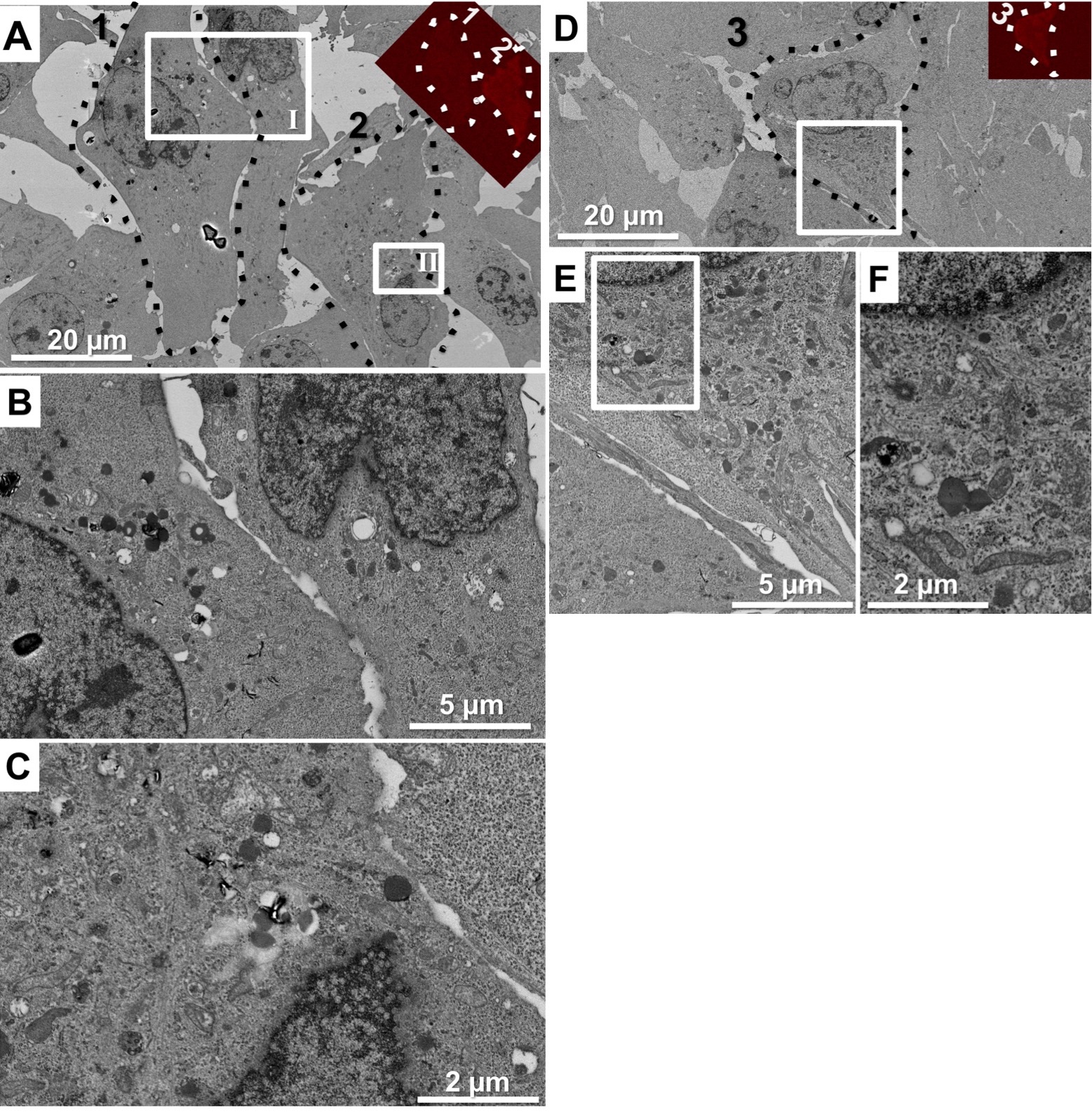


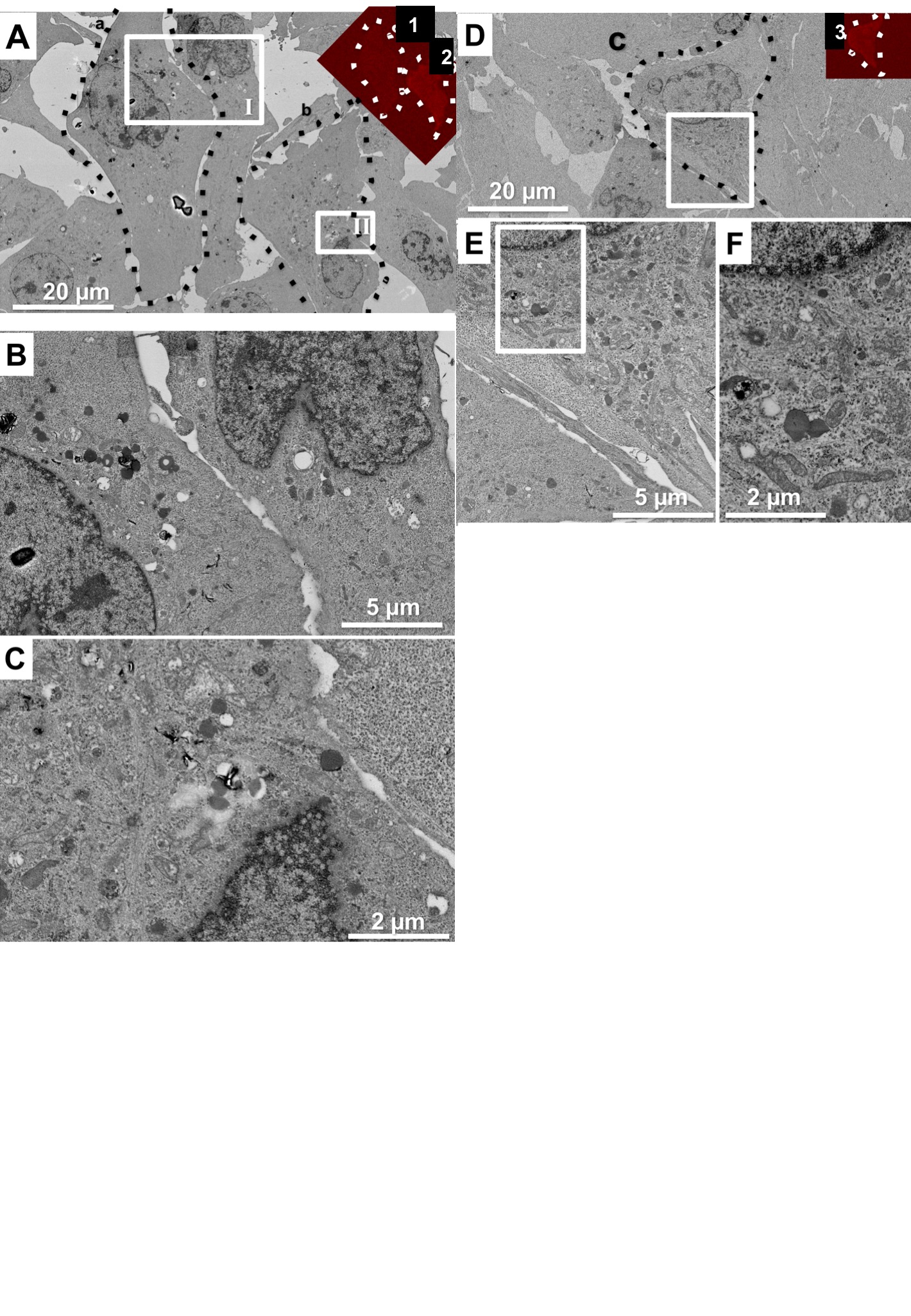
**Supplementary Fig. 2. Colocalizaiton of mKate2-mito, mKate2-Golgi, and mKate2-ER proteins with respcetive Tom20 (mitochondrial marker), GM130 (the Golgi marker), and calnexin (endoplasmic reticulumn marker).** The mKate2-mito, mKate2-Golgi, and mKate2-ER proteins were expressed in HEK293 cells. The cells were fixed with 4% papraformaldehyde, and premeabilized with phosphate buffered serum caontaining 0.1% saponin. After blocking the cells in phosphate buffered serum containing 1% BSA, rabbit polyclonal anti-Tom20 (Abcam, #ab78547, x100 dilution), anti-GM130 (GeneTex, # GTX79420, x100 dilution), and anti-calnexin antibodies (Abcam, # ab22595, x100 dilution) were incubated in a phosphate buffered serum containing 0.1% BSA. As a seconday atibody, Alexa Fluor 448-conjugated goat anti-rabbit IgGs antobody (Thermo Fisher Scientific, #A-11008, x1000 dilution) was employed. The fluorescent images were obtained with a BZ-X710 fluorescence microscope (Keyence) using GFP and Texas Red filters (CCD monochrome camera, NIKON CFI60 series x20 lens, gain +8dB). Scale bars, 20µm.


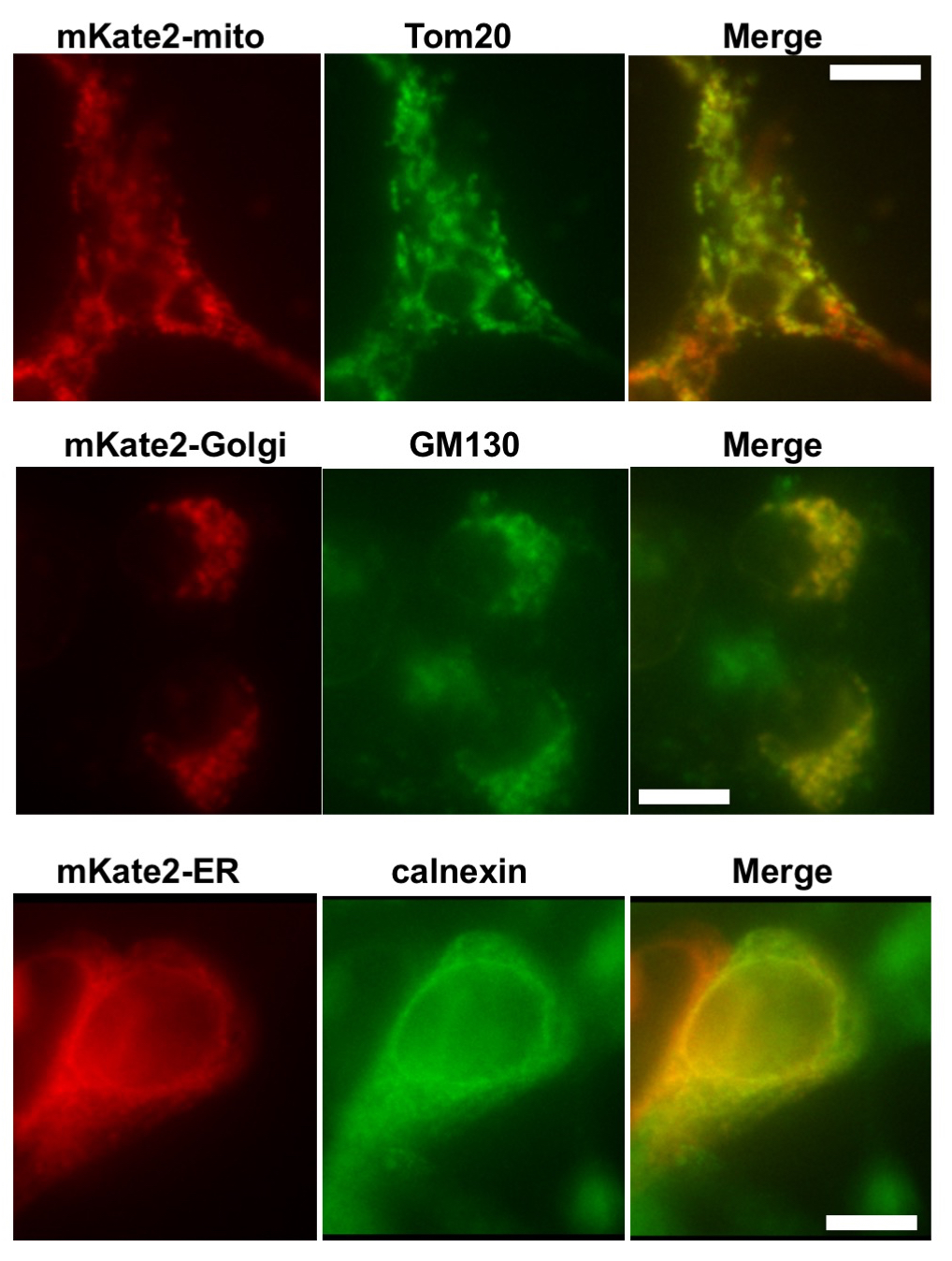


**Supplementary Fig. 3. Little fluorescence is retained in mStable, mCherry, and mApple after osmium staining.** Cells expressing each fluorescent protein were treated and detected with a Texas Red filter set under the same conditions for mKate2 as described in Supplementary Fig. 3.

**
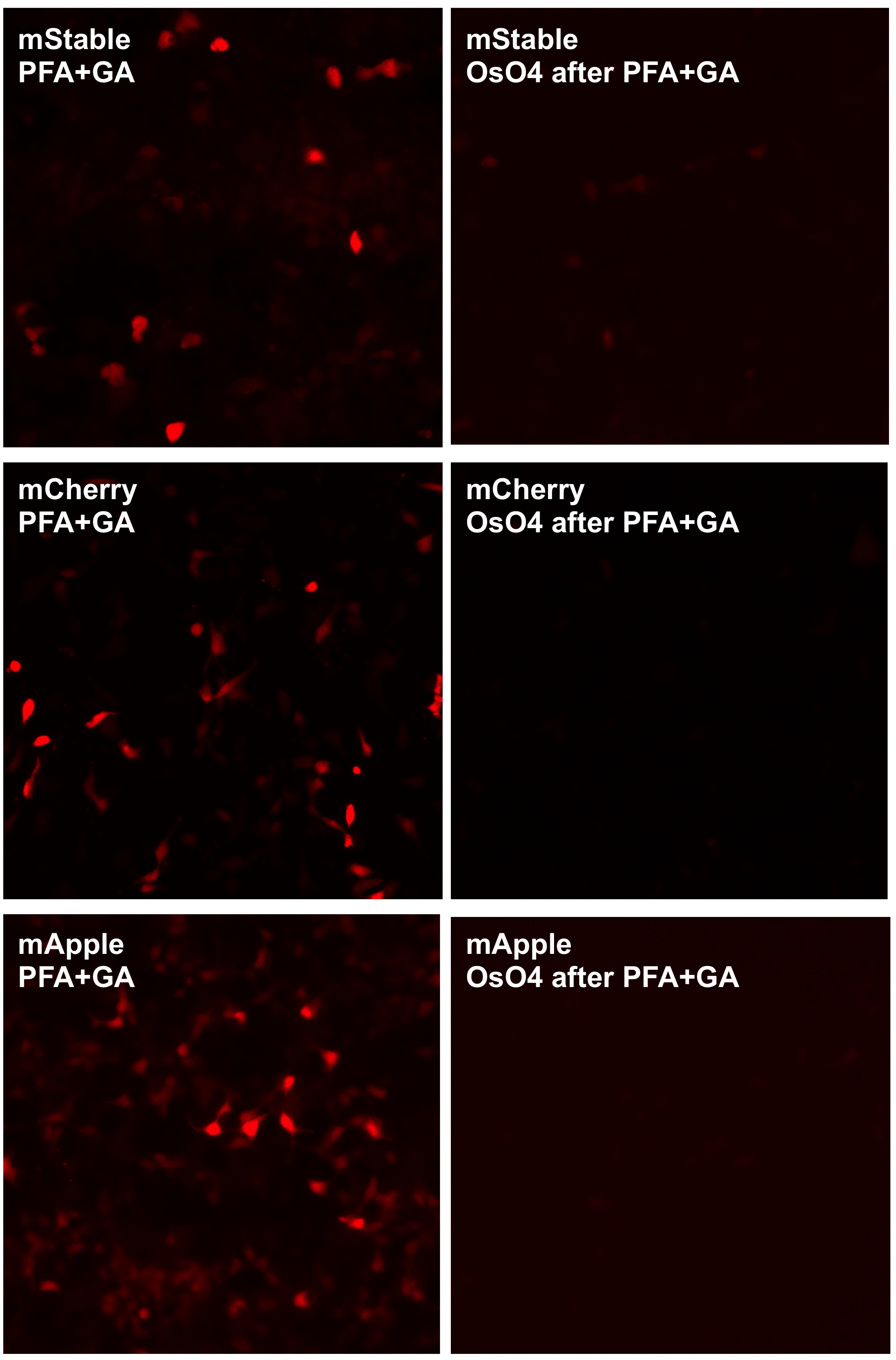
**

**Supplementary Fig. 4. Comparison of mKate2 with mEosEM after osmium staining.** For the expression of mEosEM, a mEos4b-C1 plasmid (Addgene, # #54812) was site-directed mutagenized to generate mEosEM by high fidelity PCR and Gibson assembly with six primers (mEosEM67-109F, 5’-CACTTTGTGATCGACGGAGAAGGTACAGGCAAGCCTTATGAGG-3’; mEosEM67-109Rv, 5’-CCTCATAAGGCTTGCCTGTACCTTCTCCGTCGATCACAAAGTG-3’; mEosEM256-303F, 5’- AAGGGGTATTCGTGGGAACGAAGCATGACTTTCGAAGACGGGGGCATT-3’; mEosEM256-303Rv, 5’-AATGCCCCCGTCTTCGAAAGTCATGCTTCGTTCCCACGAATACCCCTT-3’; mEosEM475-524F, 5’- GAGATGGCTTTGTTGCTTGAAGGAGGTGCCCATTACCGATGTGACTTCAG-3’; mEosEM475-524Rv, 5’-CTGAAGTCACATCGGTAATGGGCACCTCCTTCAAGCAACAAAGCCATCTC-3’) (pmEosEM-C). The pmEosEM-C and pmKate2-C (Evrogen, #FP181) was introduced into HEK293 cells, since both plasmids have the same backbone plasmid. At 24h after transfection, cells were prefixed in 2% paraformaldehyde + 2.5% glutaraldehyde (**PFA+GA**) at 4ºC for 30 min. After wahing the fixed cells with phosphate buffered serum three times, cells were postfixed with 1% osmium tetroxide at 4ºC for 5 minutes (**OsO4**). After washing the cells with phosphate buffered serum, the fluorescent images were obtained with a BZ-X710 fluorescence microscope (Keyence) using **DAPI**, **GFP** and **Texas Red** filters (CCD monochrome camera, NIKON CFI60 series x4 lens, gain +8dB, x2 digital zoom). The time (**1/30**, **1/3**, **1/8.5**, and **3** sec) in the images indicate shutter speeds of CCD camera. Scale bars, 50µm. Note that the far red fluorescence of mKate2 in the cells was detected after osmium staining with Texas Red filter (shutter speed 1/3 sec), while little green and blue fluorescence of the same cells was detected with respective GFP and DAPI filters. In contrast, the green fluorescence of mEosEM in the cells was detected after osmium staining with GFP filter (shutter speed 3 sec), but this long exposure lead to high fluorescence background of green and blue fluorescence with respective GFP and DAPI filters.


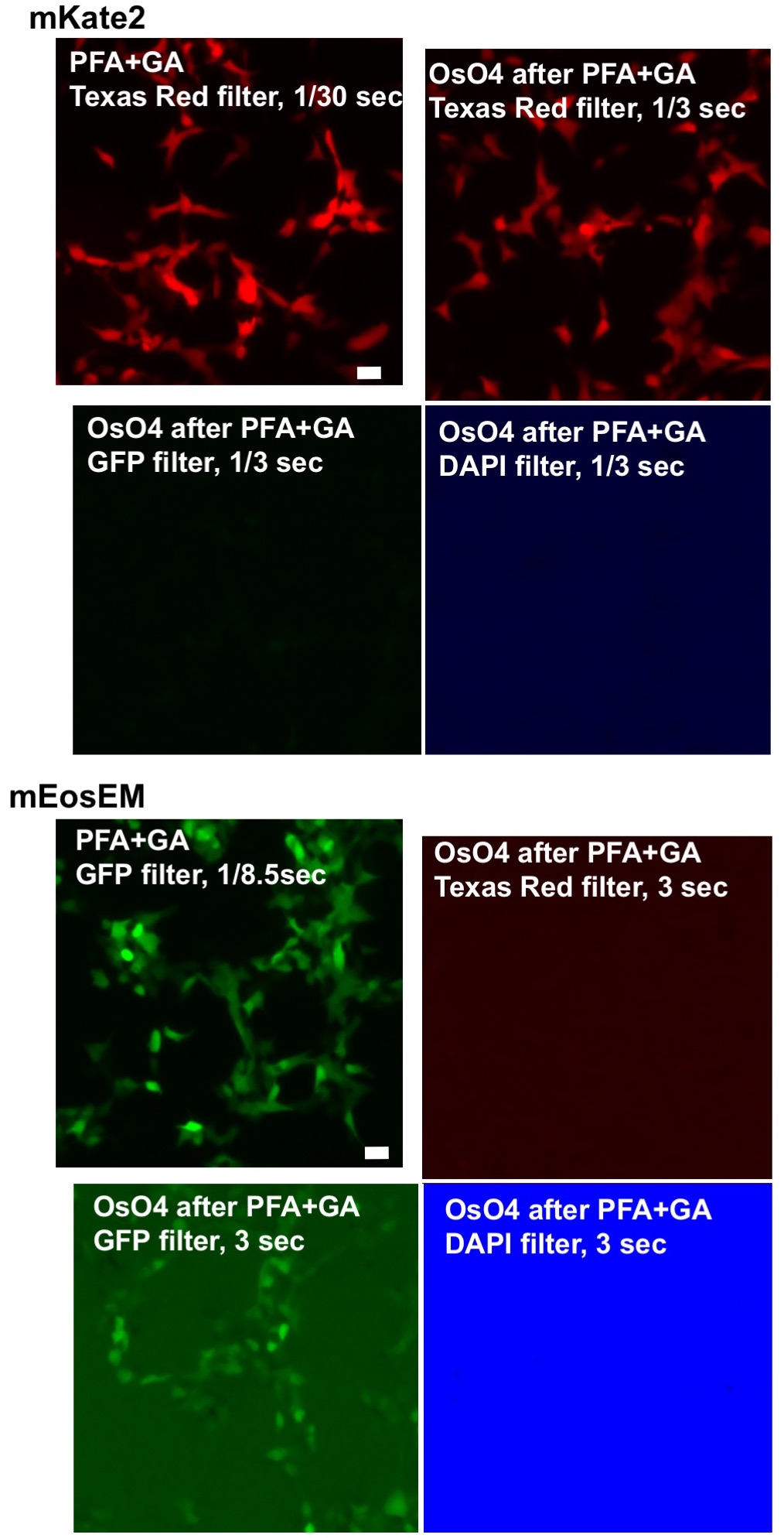


Supplementary data. The DNA sequences encoding mKate2 and its fusion proteins.

>mKate2 with GGGGSGL ATGGTGAGCGAGCTGATTAAGGAGAACATGCACATGAAGCTGTACATGGAGGGCACCGTGAACAACCACCACTTCAAGTGCACATCCGAGGGCGAAGGCAAGCCCTACGAGGGCACCCAGACCATGAGAATCAAGGCGGTCGAGGGCGGCCCTCTCCCCTTCGCCTTCGACATCCTGGCTACCAGCTTCATGTACGGCAGCAAAACCTTCATCAACCACACCCAGGGCATCCCCGACTTCTTTAAGCAGTCCTTCCCCGAGGGCTTCACATGGGAGAGAGTCACCACATACGAAGACGGGGGCGTGCTGACCGCTACCCAGGACACCAGCCTCCAGGACGGCTGCCTCATCTACAACGTCAAGATCAGAGGGGTGAACTTCCCATCCAACGGCCCTGTGATGCAGAAGAAAACACTCGGCTGGGAGGCCTCCACCGAGACCCTGTACCCCGCTGACGGCGGCCTGGAAGGCAGAGCCGACATGGCCCTGAAGCTCGTGGGCGGGGGCCACCTGATCTGCAACTTGAAGACCACATACAGATCCAAGAAACCCGCTAAGAACCTCAAGATGCCCGGCGTCTACTATGTGGACAGAAGACTGGAAAGAATCAAGGAGGCCGACAAAGAGACCTACGTCGAGCAGCACGAGGTGGCTGTGGCCAGATACTGCGACCTCCCTAGCAAACTGGGGCACAGAGGTGGAGGAGGTTCCGGACTC

>mKate2-mitochondria

ATGGTGAGCGAGCTGATTAAGGAGAACATGCACATGAAGCTGTACATGGAGGGCACCGTGAACAACCACCACTTCAAGTGCACATCCGAGGGCGAAGGCAAGCCCTACGAGGGCACCCAGACCATGAGAATCAAGGCGGTCGAGGGCGGCCCTCTCCCCTTCGCCTTCGACATCCTGGCTACCAGCTTCATGTACGGCAGCAAAACCTTCATCAACCACACCCAGGGCATCCCCGACTTCTTTAAGCAGTCCTTCCCCGAGGGCTTCACATGGGAGAGAGTCACCACATACGAAGACGGGGGCGTGCTGACCGCTACCCAGGACACCAGCCTCCAGGACGGCTGCCTCATCTACAACGTCAAGATCAGAGGGGTGAACTTCCCATCCAACGGCCCTGTGATGCAGAAGAAAACACTCGGCTGGGAGGCCTCCACCGAGACCCTGTACCCCGCTGACGGCGGCCTGGAAGGCAGAGCCGACATGGCCCTGAAGCTCGTGGGCGGGGGCCACCTGATCTGCAACTTGAAGACCACATACAGATCCAAGAAACCCGCTAAGAACCTCAAGATGCCCGGCGTCTACTATGTGGACAGAAGACTGGAAAGAATCAAGGAGGCCGACAAAGAGACCTACGTCGAGCAGCACGAGGTGGCTGTGGCCAGATACTGCGACCTCCCTAGCAAACTGGGGCACAGAGGTGGAGGAGGTTCCGGACTCACCGGTGGAGGCGGTTCAGGCGGAGGTGGCTCTGGCGGTGGCGGATCGAGATCTCGAGCTCAAGCTTCGAATTCTAAACTAATTGCTAAAAGTGCAGAAGACGAAAAAGCGAAGGAAGAACCAGGGAACCATACGATCGTAATTCTTGCAATGTTAGCTATTGGCGTGTTCTCTTTAGGGGCGTTTATCAAAATTATTCAATTAAGAAAAAATAAT

>mKate2-Golgi

ATGAGGCTTCGGGAGCCGCTCCTGAGCGGCAGCGCCGCGATGCCAGGCGCGTCCCTACAGCGGGCCTGCCGCCTGCTCGTGGCCGTCTGCGCTCTGCACCTTGGCGTCACCCTCGTTTACTACCTGGCTGGCCGCGACCTGAGCCGCCTGCCCCAACTGGTCGGAGTCTCCACACCGCTGCAGGGCGGCTCGAACAGTGCCGCCGCCATCGGGCAGTCCTCCGGGGAGCTCCGGACCGGAGGGGCCAAGGATCCACCGGTCGCCACCATGGTGAGCGAGCTGATTAAGGAGAACATGCACATGAAGCTGTACATGGAGGGCACCGTGAACAACCACCACTTCAAGTGCACATCCGAGGGCGAAGGCAAGCCCTACGAGGGCACCCAGACCATGAGAATCAAGGCGGTCGAGGGCGGCCCTCTCCCCTTCGCCTTCGACATCCTGGCTACCAGCTTCATGTACGGCAGCAAAACCTTCATCAACCACACCCAGGGCATCCCCGACTTCTTTAAGCAGTCCTTCCCCGAGGGCTTCACATGGGAGAGAGTCACCACATACGAAGACGGGGGCGTGCTGACCGCTACCCAGGACACCAGCCTCCAGGACGGCTGCCTCATCTACAACGTCAAGATCAGAGGGGTGAACTTCCCATCCAACGGCCCTGTGATGCAGAAGAAAACACTCGGCTGGGAGGCCTCCACCGAGACCCTGTACCCCGCTGACGGCGGCCTGGAAGGCAGAGCCGACATGGCCCTGAAGCTCGTGGGCGGGGGCCACCTGATCTGCAACTTGAAGACCACATACAGATCCAAGAAACCCGCTAAGAACCTCAAGATGCCCGGCGTCTACTATGTGGACAGAAGACTGGAAAGAATCAAGGAGGCCGACAAAGAGACCTACGTCGAGCAGCACGAGGTGGCTGTGGCCAGATACTGCGACCTCCCTAGCAAACTGGGGCACAGAGGTGGAGGAGGTTCCGGACTC

>mKate2-ER ATGCTGCTATCCGTGCCGTTGCTGCTCGGCCTCCTCGGCCTGGCCGTCGCCGACCGGTCGCACACCATGGTGAGCGAGCTGATTAAGGAGAACATGCACATGAAGCTGTACATGGAGGGCACCGTGAACAACCACCACTTCAAGTGCACATCCGAGGGCGAAGGCAAGCCCTACGAGGGCACCCAGACCATGAGAATCAAGGCGGTCGAGGGCGGCCCTCTCCCCTTCGCCTTCGACATCCTGGCTACCAGCTTCATGTACGGCAGCAAAACCTTCATCAACCACACCCAGGGCATCCCCGACTTCTTTAAGCAGTCCTTCCCCGAGGGCTTCACATGGGAGAGAGTCACCACATACGAAGACGGGGGCGTGCTGACCGCTACCCAGGACACCAGCCTCCAGGACGGCTGCCTCATCTACAACGTCAAGATCAGAGGGGTGAACTTCCCATCCAACGGCCCTGTGATGCAGAAGAAAACACTCGGCTGGGAGGCCTCCACCGAGACCCTGTACCCCGCTGACGGCGGCCTGGAAGGCAGAGCCGACATGGCCCTGAAGCTCGTGGGCGGGGGCCACCTGATCTGCAACTTGAAGACCACATACAGATCCAAGAAACCCGCTAAGAACCTCAAGATGCCCGGCGTCTACTATGTGGACAGAAGACTGGAAAGAATCAAGGAGGCCGACAAAGAGACCTACGTCGAGCAGCACGAGGTGGCTGTGGCCAGATACTGCGACCTCCCTAGCAAACTGGGGCACAGAGGTGGAGGAGGTAGATCGTACAAGAAGGACGAGCTG
